# A Sequential Therapeutic Hydrogel With Injectability and Antibacterial Activity for Deep Burn Wounds’ Cleaning and Healing

**DOI:** 10.3389/fbioe.2021.794769

**Published:** 2021-12-02

**Authors:** Sizhen Wang, Jie Li, Zhiqiang Ma, Linhong Sun, Lei Hou, Ying Huang, Yunchang Zhang, Beibei Guo, Feng Yang

**Affiliations:** ^1^ College of Pharmacy, Fujian University of Traditional Chinese Medicine, Fuzhou, China; ^2^ Department of Dermatology, 967 Hospital of the Joint Logistics Support Force of PLA, Dalian, China; ^3^ Department of Inorganic Chemistry, School of Pharmacy, Second Military Medical University, Shanghai, China

**Keywords:** hydrogel, sequential therapy, deep burn wounds, eschar cleaning, antibiosis, injectability

## Abstract

As a severe clinical challenge, escharotomy and infection are always the core concerns of deep burn injuries. However, a usual dressing without multifunctionality leads to intractable treatment on deep burn wounds. Herein, we fabricated a sequential therapeutic hydrogel to solve this problem. Cross-linked by modified polyvinyl alcohol (PVA-SH/ε-PL) and benzaldehyde-terminated F127 triblock copolymers (PF127-CHO), the hydrogel demonstrated excellent mechanical properties, injectability, tissue adhesiveness, antibacterial activity, biocompatibility, and satisfactory wound cleaning through both *in vitro* and *in vivo* assays. Additionally, based on the conception of “sequential therapy,” we proposed for the first time to load bromelain and EGF into the same hydrogel in stages for wound cleaning and healing. This work provides a strategy to fabricate a promising wound dressing for the treatment of deep burn wounds with injectability and improved patients’ compliance as it simplified the process of treatment due to its “three in one” characteristic (antibacterial activity, wound cleaning, and healing effects); therefore, it has great potential in wound dressing development and clinical application.

## Introduction

Burn injury is a major cause of trauma in both civilian and military environments (Yaacobi et al., 2021). Each year, there are more than 11 million burn patients with severe necrosis, and about 180,000 people died (Najath et al., 2021; Kaddoura et al., 2017; Dong et al., 2020). Also, the complex wound environment leads to a series of complications that occur during the long and slow repair processes. The necrosis and complication gravely threaten to burn patients’ lives. Traditional treatment for burn injuries includes debridement, skin grafting for large-scale burn, and medical care to prevent infection and promote wound healing. Hence, it always takes a long period to heal the burn wound, especially for the debridement surgery in which parts of healthy tissue are inevitably discarded, generating a more complicated and aggravated situation. Besides, in a medically deprived environment, deficiency of medical care makes debridement surgery unavailable and a lower compliance of burned patients. In short, the above situations urgently call a simple and effective treatment for burn injuries.

A number of products have been utilized to dress the burn wounds with simpler and more efficient strategies. For example, NexoBrid® from Israel is a selective enzymatic wound cleaning product (Rosenberg et al., 2014; Shoham et al., 2020). It can clean eschar based on bromelain without surgery in clinical research, which has been included in the European Consensus Guidelines since 2017 (Hirche et al., 2020). Unfortunately, without injectability, insufficient coating may occur on the irregular shaped wounds with NexoBrid®. Besides, gauze (Fang et al., 2021; Chen et al., 2021a; Said et al., 2021), electrospun nanofiber (Lutfi et al., 2021; Shahrousvand et al., 2021; Zhu et al., 2021), porous foam (Wang et al., 2008; Staruch et al., 2017), biocompatible membrane (Huang et al., 2021), microfibers (Zhao et al., 2015; Yu et al., 2017), and hydrogels (Soriano et al., 2020; Yuan et al., 2021; Kopecki, 2021) can be used as burn wound dressings today. However, most of them cannot display critical properties such as exudates which are absorbing and moisture-maintaining at the same time compared with hydrogel dressings (Gou and Ma, 2018; Zhao et al., 2018; Gobhril and Grinstaff, 2015). Last but not least, burn injury is always intractable as a result of infection, so dressings with antimicrobial activity are requisite (Chen et al., 2021b). Therefore, injectable dressing with exudates absorbing, moisture-maintaining, and antibacterial effect is urgently demanded for deep burn wound.

Herein, we fabricated a novel multifunctional hydrogel with a triple-network structure for wound cleaning and accelerating burn wound healing. Briefly, precursor solutions such as PF127-CHO and PVA-SH/ε-PL were mixed and stirred to make PVA-SH/ε-PL-az-PF127-CHO hydrogel (PLC). In this system, PVA-SH was designed to prevent bromelain oxidation by the sulfhydryl groups. Moreover, amino groups on PVA-SH and ε-PL activated Schiff-base reaction with PF127-CHO to provide a double-network structure; at the same time, PF127-CHO was self-assembled into micelles through physical cross-linking, leading to the preparation of triple-network PLC hydrogel. Meanwhile, partial sulfhydryl groups on PVA-SH could be oxidized to form disulfide bonds to enhance cross-linking. The novel Schiff-base hydrogel we designed had the inherent ability of an antibacterial, which was credited to Schiff-base compounds (Huijun et al., 2020; Suo et al., 2021). What is more, the weak acid environment of the wound promotes the release of ε-PL from PLC, improving the antibacterial efficiency of PLC because of its fantastic antimicrobial property (Huang et al., 2020). The anti-infective effect against methicillin-resistant *Staphylococcus aureus* (MRSA) and *Escherichia coli* (*E. coli*) was verified by antibacterial assays *in vitro*, and the tissue adhesiveness was confirmed by the adhesive strength test. Based on a concept of “sequential therapy” first proposed here, which means different treatments are adopted at certain times in a course of treatment, we loaded bromelain (BM) and EGF in stages to prepare (BM/EGF)@PLC at a certain time for wound cleaning and healing. As a result, with its injectability, (BM/EGF)@PLC is geared for irregular shaped wounds; with the combination of “sequential therapy,” (BM/EGF)@PLC shows great potential in dressing development and clinical treatment for deep burn wounds while improving patients’ compliance.

## Materials and Methods

### Materials

Polyvinyl alcohol (PVA) and Pluronic F127 (PF127, Mn = 12,600) were purchased from Sinopharm Chemical Reagents Co. Ltd. (China) and BASF(Germany), respectively. Glutathione (GSH), L-Cysteine (L-Cys), ethyl sulfonyl chloride, triethylamine, p-hydroxy benzaldehyde, and bromelain were supplied by damas-beta (China). ε-poly-L-lysine (ε-PL) and human recombinant epidermal growth factor (EGF) were provided by Alladin (China) and Shanghai Huidun Biotechnology Co., Ltd (China), respectively.

### Preparation of PLC Hydrogels and Drug-Loaded Hydrogels

Methods on the preparation of hydrogel precursors were described in the Supplementary Material. PLC hydrogels and drug-loaded hydrogels were prepared as below: PF127-CHO was dissolved in phosphate buffer saline (PBS) (10 mM, pH 7.4) to a final concentration of 25% (w/v). PVA-SH/ɛ-PL solution was prepared according to the molar ratio of carboxyl groups on ɛ-PL and hydroxy groups on PVA, which were 75:1, 50:1, 100:1, and 200:1, respectively. Then, PF127-CHO and PVA-SH/ɛ-PL solution was mixed homogenously with the molar ratio of aldehyde groups on PF127-CHO and amino groups on PVA-SH/ɛ-PL being 3:1, and was stirred for complete hydrogel formation. The obtained hydrogels were named according to the molar ratio of the precursor solutions. Briefly, the hydrogel prepared with PF127-CHO and PVA-SH/ɛ-PL (75:1, 50:1, 100:1, and 200:1) was named PLC3, PLC3-1, PLC3-2, and PLC3-3, respectively. To encapsulate bromelain and EGF into the hydrogel in stages, bromelain and EGF aqueous solution containing the pre-set amounts of drugs were mixed with PF127-CHO solution and then hydrogel was formed with PVA-SH/ɛ-PL, respectively. The final drug-loaded hydrogel system was simplified as (BM/EGF)@PLC.

### Characterization

Polymers were verified by ^1^H NMR, and the special functional groups were recorded by FT-IR. SEM (Tescan Mira 3 XH, Czech Republic) was utilized to investigate the morphology of PLC after freeze-drying. Rheological properties of PLC were studied by a rheometer (HAAKE MARS, thermo scientific). The properties of swelling, degradation, and moisturizing were assessed as below.

For the swelling test, the freeze-dried hydrogel samples were weighed accurately and immersed in PBS (pH 7.4) solution at 37°C. Then, the samples were taken out and weighed immediately after drying the surface. The swelling ratio (SR) was calculated by the following equation:

$$SR\left(\%\right)=\frac{\left(W_{t}-W_{0}\right)}{W_{0}}\times 100\%,$$

where W_t_ is the weight of PLC at predetermined time intervals and W_0_ is the weight of the initial dried sample.

For the degradation kinetics, hydrogel bulks were immersed into PBS (pH7.4) and incubated at 37°C. At predetermined time intervals, the hydrogel samples were taken out and freeze-dried before the determination of dry weight. The biodegradation rate (BR) was calculated according to the following equation:

$$BR\left(\%\right)=\frac{\left(W_{0}-W_{t}\right)}{W_{0}}\times 100\%,$$

where W_0_ and W_t_ are the weights of original hydrogels and remanent hydrogels, respectively.

For the moisture retention assay, PLCs were weighed before being put into the dryer and then taken out to be weighed at the predetermined time. The moisture rate (MR) was calculated by the following equation:

$$MR\left(\%\right)=\frac{\left(W_{t}-W_{0}\right)}{W_{0}}\times 100\%,$$

where W_t_ is the weight of PLC at predetermined time intervals and W_0_ is the weight of the initial sample. The experiments were conducted in triplicate.

### *In Vitro* Drug Release Assay

1 ml PLC hydrogel loaded with bromelain (10 mg) were immersed into 5 ml PBS (pH = 7.4) and placed into a shaking incubator of 37°C, 100 rpm. At pre-set intervals, 60 μL release medium was withdrawn and 60 μL fresh buffer was supplemented. The absorbance of the release medium at 562 nm was measured. The release amount of bromelain was calculated using a predetermined standard profile. The *in vitro* release of EGF (3 mg) from the hydrogel was conducted in PBS using the aforementioned method. The release amounts of both drugs were quantified with a BCA Protein Assay Kit (P0012, Beyotime).

### Adhesive Strength Test

Adhesive strength of PLC was tested by a lap shear testing (Li et al., 2018). Briefly, 0.3 ml hydrogel precursor solution of PLC (PLC1, PLC3, PLC3-1, PLC3-2, and PLC3-3) was applied onto a piece of gelatin-coated glass to form a hydrogel layer (PLC1 was synthesized with a similar method as PLC3, except for the use of PF127 instead of PF127-CHO in preparation). After that, the sample was pasted by another piece of gelatin-coated glass with a contacting area of 25 mm × 25 mm and tested by a tensile machine (TH-8203A). All measurements were conducted in triplicate.

### Antimicrobial Test

The antibacterial activity of PLC was tested by the inhibitor zone test and agar plate colony counting test against MRSA and *E. coli*. First, the inhibitor zone test was performed with bacteria suspension applied on the culture medium. Second, hydrogel samples were cut into about 10 mm in diameter and put on the surface of the culture medium to cover the bacteria, and then cultured at 37°C for 24 h. The antibacterial effect was assessed by measuring the diameter of the inhibitor zone. For the agar plate colony counting test, the hydrogel extract was added into bacteria suspension and the mixture was applied on the culture medium evenly, and then cultured at 37°C for 24 h. The number of colonies was counted to evaluate the antibacterial activity of PLC. All measurements were conducted in triplicate.

### Cytocompatibility Evaluation

The cytocompatibility of PLC was evaluated by the following tests. Briefly, L929 cells were seeded into 96-well microplates at a density of 1×10^4^ cells per well. After incubation for 12 h, 100 μL culture medium containing different concentrations (10, 5, 2.5, 1, and 0.5 mg/ml) of the hydrogel extracts (PLC3, PLC3-1, PLC3-2, and PLC3-3) was added into per well and incubated for 24 h, respectively. After washing with PBS, CCK-8 dye solution was used to evaluate the cell viability.

The Live/Dead assay and CCK-8 assay were further conducted to assess the cytocompatibility of (BM/EGF)@PLC including BM@PLC3 and EGF@PLC3. CCK-8 assay was conducted in the same way as the method above, except the concentrations (10, 5, 2.5, 1.25, and 0.625 mg/ml) of hydrogel samples (PLC3, BM@PLC3, and EGF@PLC3). The Live/Dead staining assay was carried out with L929 cells seeded into 24 well microplates at a density of 5 ×10^4^ cells per well. After incubation for 12 h, the 300 μL culture medium containing EGF@PLC3 (10 μg/ml of EGF), EGF (10 μg/ml), BM@PLC3 (20 μg/ml of bromelain), and bromelain (20 μg/ml) was added into per well, respectively, and incubated for 24 h. The fluorescent images were taken by a fluorescence microscope, on which live cells were stained as green and dead cells were stained as red. All measurements were conducted in triplicate.

### Hemolytic Test

Briefly, the hydrogel extract (PLC3, PLC3-1, PLC3-2, PLC3-3, BM@PLC3, and EGF@PLC3) and the red blood cell suspension were mixed with equal volumes and incubated at 37°C for 1 h. The samples were centrifugated and the supernatants were collected to determine the absorbance value under 540 nm. Besides, the sodium chloride injection (0.9%) and the red blood cell suspension that mixed with equal volume served as the negative group, while using ultra-pure water instead of sodium chloride injection (0.9%) served as the positive group. The hemolysis ratio (HR) was calculated by the following equation:

$$HR\left(\%\right)=\frac{\left(A_{i}-A_{n}\right)}{\left(A_{p}-A_{n}\right)}\times 100\%,$$

where A_i_ is the absorbance value of hydrogel samples, and A_n_ and A_p_ are the absorbance values of negative and positive groups, respectively. The experiment was conducted in triplicate.

### Cell Scratch Experiment

L929 cells were seeded into 6-well microplates at a density of 4×10^5^ cells per well. After incubation for 48 h, 2 ml culture medium containing EGF@PLC3, EGF, and PLC3 was added into per well, respectively. The result in each group was investigated by a microscope, and photos were taken at 0, 24, and 48 h, respectively. The scratch area in each group was measured by ImageJ. The migration rate (MR) was calculated by the following equation:

$$MR\left(\%\right)=\frac{\left(S_{0}-S_{i}\right)}{S_{0}}\times 100\%,$$

where S_i_ is the scratch area of the hydrogel at predetermined time intervals and S_0_ is the area of 0 h. The experiment was conducted in triplicate.

### Deep Partial-Thickness Skin Burns’ Wound Healing Effect *In Vivo*

Male Sprague Dawley rats (SD), weighing 200–250 g, were divided into 4 groups randomly, with 6 rats in each group. A deep partial-thickness skin burn wound on the dorsum was made using a scald apparatus (ZH-YLS-5Q, Huaibei Zhenghua Biological Instrument Co., Ltd) with a copper rod (11 mm, 100°C, 10 s) after anesthetizing by intraperitoneal injection of 10% chloral hydrate at a dose of 0.3 ml/100 g. Then, the (BM/EGF)@PLC3 group was covered with (BM/EGF)@PLC3 hydrogels in stages according to the “sequential therapy.” Therein, BM@PLC3 was used on the first day, while EGF@PLC3 was adopted in the following treatment. The PLC3 group was treated with PLC3 dressing, the positive group was covered with sulfadiazine silver cream (25 g, China Resources Kunming Shenghuo Pharmaceutical Co., Ltd.), and the control group with no further treatment. The dressings were renewed every day, and the burn wounds were photographed on days 0, 2, 8, 15, and 21 with a digital camera before treatment. Besides, the wound closure rate (WR) was described by the following equation:

$$WR\left(\%\right)=\frac{\left(W_{0}-W_{i}\right)}{W_{0}}\times 100\%,$$

where W_0_ is the initial wound area, and W_i_ is the residual wound area on days 2, 8, 15, and 21 before treatment.

### Histological Analysis

The wound tissue samples were collected on days 0, 8, 15, and 21 before treatment and fixed in 4% formaldehyde solution before being embedded in paraffin, following by cutting into 3 mm sections. Afterward, samples were tested with H&E, Masson’s trichrome, and immunohistochemical staining (CD31, VEGF).

### The Effect of Removing Eschar on a Full-Thickness Skin Burn Model

16 rats were divided into 4 groups randomly, including BM@PLC3 group, PLC3 group, positive group, and control group. Then, a full-thickness skin burn wound was made by the scald instrument with 100°C for 20 s. The wound was treated with different dressings as above, and photos were taken on days 0, 3, 5, and 12. The wound tissues were collected on days 0, 5, and 12 before treatment and fixed in 4% formaldehyde solution to carry out H&E.

### Statistical Analysis

The statistical significance was analyzed by statistical computation using SPSS (Version 23.0). Briefly, one-way ANOVA was used for three or more groups. Data were presented as mean ± SD. The significant level was set as *p* < 0.05.

## Results and Discussions

### Structural Characterization and Rheological Properties

The preparation routes of PF127-CHO/ɛ-PL, PVA-SH, and PLC hydrogel are shown in Supplementary Figure S1, and the morphology of hydrogel is shown in Supplementary Figure S2. The results of ^1^H NMR are shown in Supplementary Figure S3; therein, the chemical shifts (*δ* = 9.87 and 9.77, *δ* = 8.67) belonged to aldehyde groups and amino groups, respectively, indicating the successful synthesis of PF127-CHO and PVA-SH. For FT-IR, the wavenumbers of 1,692.29 and 843.56 cm^−1^ belonged to the vibration of aldehyde groups on PF127-CHO; the wavenumbers of 3,069.14, 2,930.60, 1,671.43, and 1,319.79 cm^−1^ were affiliated to the vibration of carboxyl groups on PVA-SH/ɛ-PL; and the wavenumbers of 1,679.60 and 1,510.87 cm^−1^ belonged to amido groups on PLC, demonstrating the successful synthesis of PLC (Supplementary Figure S4). Above all, it could be confirmed that PLC was cross-linked through amido bonds to provide a double-network structure by dynamic Schiff-base reaction among PVA-SH, ɛ-PL, and PF127-CHO. At the same time, PF127-CHO could self-assemble to make a triple-network hydrogel, while partial sulfhydryl groups on PVA-SH could be oxidized to form disulfide bonds to enhance cross-linking.

**FIGURE 1:**
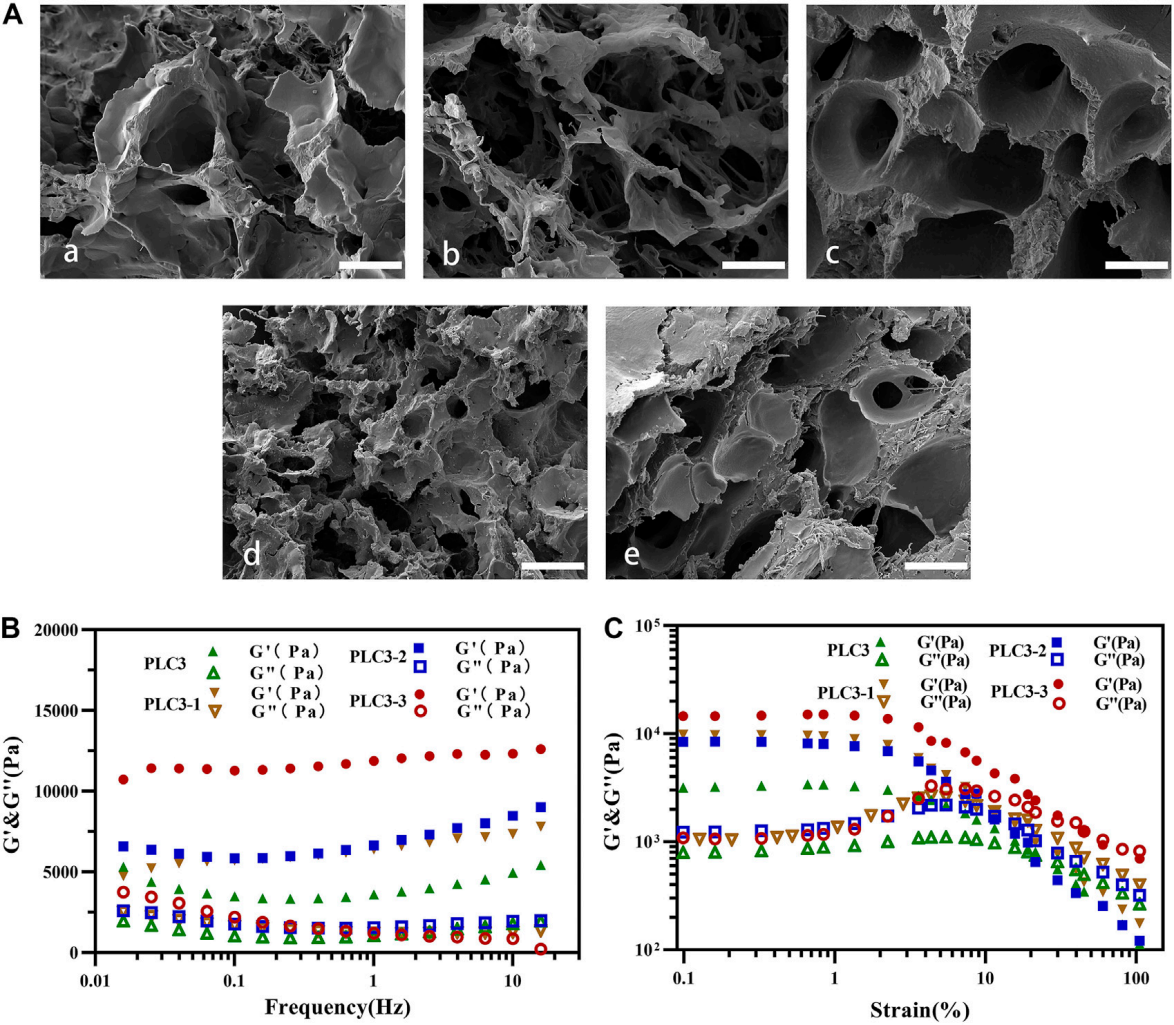
SEM investigation for **(A)** a) blank hydrogel (without ɛ-PL), b) PLC3-3, c) PLC3-2, d) PLC3, and e) PLC3-1; scale bar: 20 μm. Rheological results of PLC3, PLC3-1, PLC3-2, and PLC3-3 for **(B)** frequency sweep tests and **(C)** strain sweep tests.

SEM investigation showed the porous structures clearly, and the pore sizes of PLC hydrogels were decreased with the addition of ε-PL (Figure 1A). It obstructed the Schiff-base reaction between PF127-CHO and PVA-SH on the one hand; on the other hand, it could form a Schiff-base bond with PF127-CHO to promote cross-linking of hydrogel. Under the influence of both, the pore size of PLC was decreased.

According to the frequency sweep test (Figure 1B), the elastic modulus (G′) of these four hydrogel samples was in the range from 1,000 Pa to 10,000 Pa with a frequency from 0.01 to 10 Hz, which were reached to the modulus of natural skin tissue, suggesting their suitability of treatment for human skin (Li et al., 2018). Strain sweep test (Figure 1C) showed that the strain curves of G’ and G” in each group had similar intersection points. With the further increase of strain to 100.0%, G’ of these hydrogels decreased dramatically, due to the collapse of the hydrogel networks. Among these four samples, PLC3 had the least loss modulus, indicating that it had the most stable structure among these four samples.

### Injectable and Thermal-Responsive Performances of PLC

**FIGURE 2:**
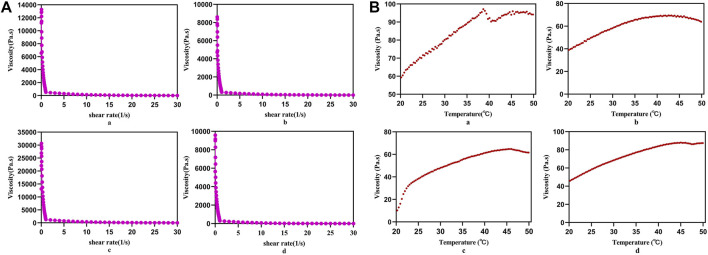
**(A)** Static shear rate sweep test and **(B)** temperature sweep test of a) PLC3, b) PLC3-1, c) PLC3-2, and d) PLC3-3.

With the increase of the shear rate, the viscosities of PLC3, PLC3-1, PLC3-2, and PLC3-3 decreased sharply (Figure 2A), showing that PLC had the shear thinning property. When the shearing rate increased to 20 s^−1^, the viscosities of the samples were all bellow 100 Pa s, implying they were quite injectable. When the temperature went up from 20 to 50°C, the viscosity increased gradually and became stable finally (Figure 2B), demonstrating that PLC had the characteristic of thermal-responsiveness. Besides, the macroscopic phenomenon showed that PLC was in the liquid form at room temperature, but when it was injected by a syringe on a heating plate under a temperature of 37°C, the hydrogel precursor solution transferred to hydrogel immediately (Supplementary Figure S4C), further indicating the injectability and thermal responsiveness of PLC. Therein, the injectable property was obtained owing to the disassociation of self-assembly PF127 micelles in the gel network.

### Self-Healing and Enhanced Adhesiveness Properties of PLC

**FIGURE 3:**
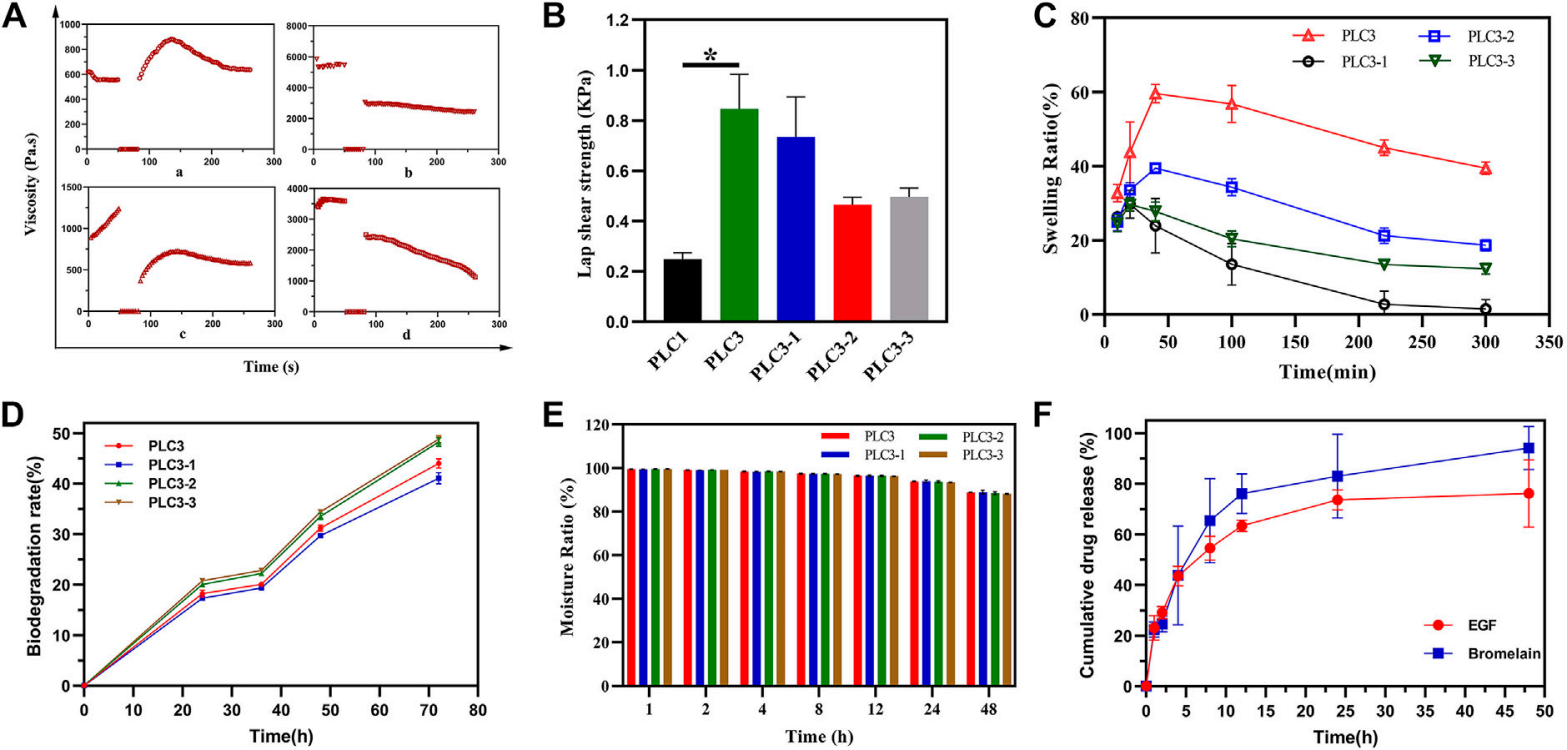
**(A)** Thixotropy test of a) PLC3, b) PLC3-1, c) PLC3-2, and d) PLC3-3. **(B)** The lap shear strength for hydrogels adhering to gelatin film. **(C)** Swelling tests, **(D)** biodegradation evaluation, and **(E)** moisture retention assay of PLC3, PLC3-1, PLC3-2 and PLC3-3. **(F)** Drugs release curve in 48 h. With the data shown as means ± SD (*n* = 3, *p < 0.05).

The thixotropy test was conducted to assess the self-healing property of PLC after external damage (Figure 3A). In the first stage, the shear rate was kept at 0.1 s^−1^ for 50 s. In the second stage, it was at 100 s^−1^ for 30 s, and then at 0.1 s^−1^ for 180 s in the end. After 40 s in the response stage (the third stage), the viscosities of PLC3, PLC3-1, PLC3-2, and PLC3-3 were restored to 151.48, 54.14, 55.73, and 64.94%, respectively. In addition, compared to the initial state, the viscosities of PLC3-1, PLC3-2, and PLC3-3 decreased by 3,019 Pa s, 651.1 Pa s, and 2,460 Pa s respectively, while that of PLC3 increased by 80.98 Pa s. The result indicated the excellent self-healing property of PLC3.

As the result of shear adhesive strength (Figure 3B), the lap shear strength of PLC3 was the strongest among these four hydrogels, and there was a significant difference between PLC1 and PLC3 (*p* < 0.05), indicating that the adhesion of PLC3 with skin tissue was promoted remarkably. The reason was that the modification of aldehyde functional groups in hydrogel system contributed to chemical cross-linking with skin tissue based on the Schiff-base reaction, leading to enhanced tissue-adhesiveness.

### Liquid Absorption, Biodegradation, and Moisturizing Performance of PLC

Liquid absorption property, one of the most important indexes for wound dressing, was evaluated by the swelling test. As shown in Figure 3C, PLC3, PLC3-1, PLC3-2, and PLC3-3 achieved the maximum swelling ratio in 50 min and then gradually descended. Among these hydrogels, PLC3 possessed the best liquid absorption performance (59.62%). This result may be owed to the components. Briefly, the molar ratio of hydroxy groups on PVA and carboxyl groups on ε-PL were 75:1, 50:1, 100:1, and 200:1 to make up PLC3, PLC3-1, PLC3-2, and PLC3-3, respectively. So, with the increase of ε-PL, the pore of these hydrogels had changed from large to small because ε-PL led to a gel block between PVA-SH and PF127-CHO; at the same time, ε-PL could gel with PF127-CHO based on the Schiff-base reaction, leading to the increased number of pores that promoted swelling. Under the double actions, PLC3 had the better swelling ratio among these samples. Biodegradability is noticeable for biosafety materials. For PLCs, the degradation performance was eligible. As shown in Figure 3D, the biodegradation rates of PLCs were all more than 40% after 72 h, indicating that PLCs possessed excellent biodegradability and there was little harm to skin. However, PLC3-2 and PLC3-3 possessed stronger biodegradability than PLC3 and PLC3-1. This was mainly because of the different cross-linking strength of PLCs. SEM showed that the pores of PLC3 and PLC3-1 were smaller than those of PLC3-2 and PLC3-3, suggesting that the cross-linking strength of PLC3 and PLC3-1 was stronger than that of PLC3-2 and PLC3-3, resulting in their lower degradation rates. Moreover, hydrogels possessed a water conservation function, which could make a hydrated environment around the wound and further facilitate the wound healing. According to Figure 3E, the moisturizing ratios of PLCs were up to 88%, revealing their excellent moisturizing performance to maintain the hydrated environment for the wound.

### Drugs Release *In Vitro*

Subsequently, the drug release behavior *in vitro* was studied. Bromelain and EGF in PLC hydrogel showed slight sudden release of 22.38 and 23.07% at 1 h, respectively. After 24 h, the cumulative release of bromelain was 82.97% and it was still releasing at a certain rate. At 48 h, the cumulative release was up to 94.14%. Different from bromelain, the cumulative release of EGF was 73.65% at 24 h, and it maintained a slow release to 48 h (76.16%) (Figure 3F). The release of the therapeutic drug was mainly mediated by the diffusion and degradation of hydrogel skeleton. However, EGF had significantly slower and longer release kinetics than bromelain, which may be due to the dynamic involvement of free amino groups in the EGF structure by the Schiff-base reaction, resulting in covalent binding of EGF to hydrogel network, thus prolonging the EGF release process. Sequential release of bromelain and EGF was very beneficial to burn wound healing. Briefly, the early release of bromelain could remove the wound eschar in time, while the long-term release of EGF effectively supported the migration and proliferation of cells, and the formation of extracellular matrix in the later stage, thus promoting wound healing.

### Antimicrobial Activity *In Vitro*

**FIGURE 4:**
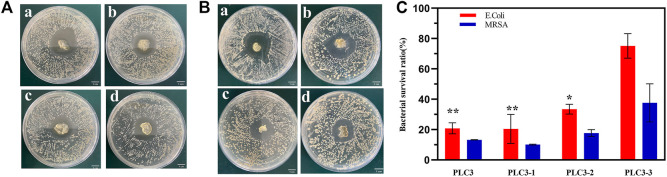
The inhibitor zone test against **(A)** *E. Coli* and **(B)** MRSA on a) PLC3-1, b) PLC3, c) PLC3-2, and d) PLC3-3; scale bar: 1 cm. **(C)** Agar plate colony counting test against *E. Coli* and MRSA on PLC3, PLC3-1, PLC3-2 and PLC3-3, with the data shown as means ± SD (*n* = 3, **p < 0.01, *p < 0.05).

The inhibitor zone test was utilized for qualitative analysis of the antibacterial property of PLC. The result (Figures 4A,B) showed that the diameters of the antibacterial zone against *E. coli* were 2.45 ± 1.06 cm (PLC3), 2.6 ± 0.42 cm (PLC3-1), 2.05 ± 1.06 cm (PLC3-2), and 2.05 ± 0.78 cm (PLC3-3), respectively, while those against MRSA were 2.5 ± 0.71 cm (PLC3), 2.6 ± 0.85 cm (PLC3-1), 2.1 ± 0.56 cm (PLC3-2), and 1.95 ± 0.64 cm (PLC3-3). The results revealed that PLC possessed antibacterial activity against *E. coli* and MRSA that depended on concentration. The agar plate colony counting test was further exploited for quantitative analysis. As shown in Figure 4C, for *E. coli*, there was significant difference among PLC3, PLC3-1, and PLC3-3 (*p* < 0.01). For MRSA, there was no significant difference between the groups, but the antibacterial activity of PLC was improved with the increased content of ε-PL. The antibacterial property was acquired on the basis of Schiff bases and further improved with the introduction of ε-PL. Briefly, PVA-SH and ε-PL were made to react with PF127-CHO based on the Schiff-base reaction; when this dressing coated on infectious wound where the environment was weak acid, the Schiff-base bonds cracked and ε-PL would release from PLC, promoting the antibacterial activity of PLC. More importantly, this hydrogel did not contain any antibiotics, and thus it had great potential for the treatment of multidrug-resistant bacterial infections according to the antibacterial test against MRSA, and it would not have any problems about microbial resistance.

### *In Vitro* Biocompatibility and Cell Proliferation Ability

**FIGURE 5:**
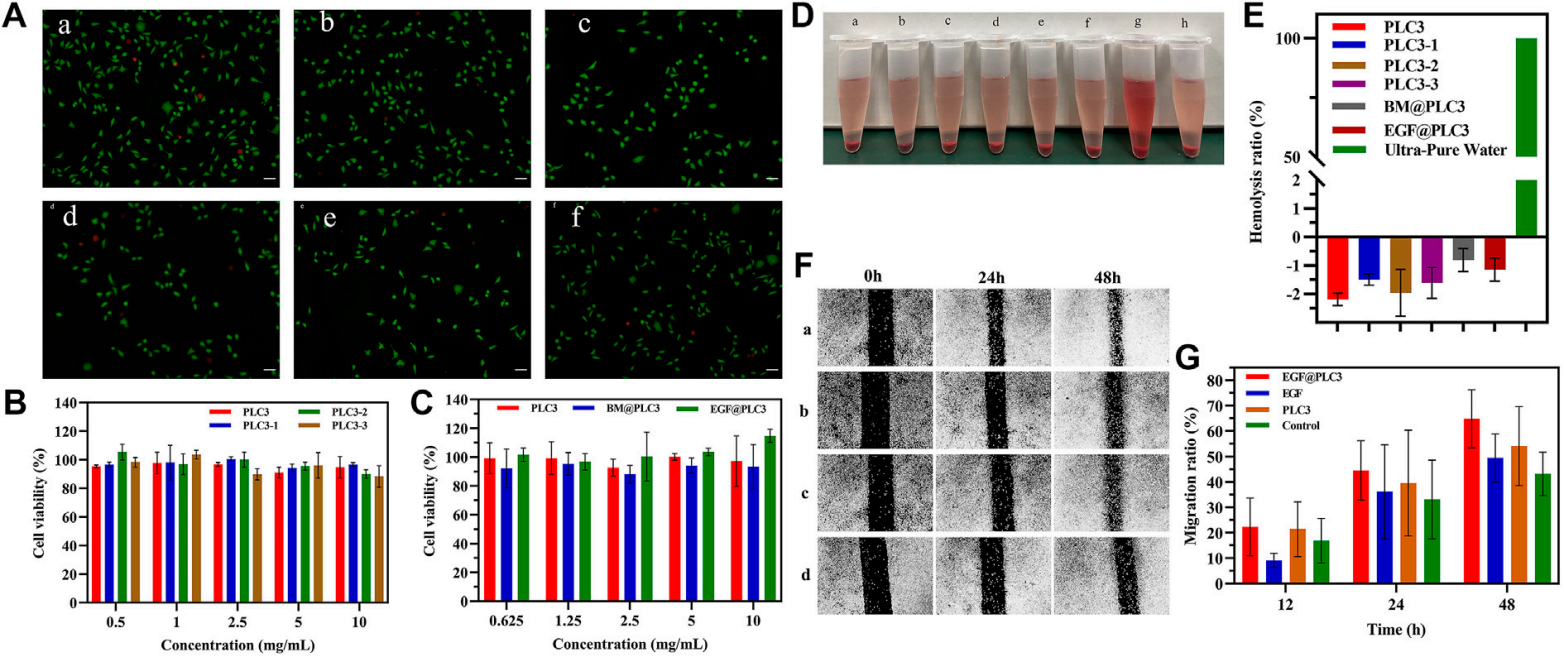
*In vitro* biocompatibility and cell-proliferation tests. **(A)** Live/Dead staining of a) EGF@PLC3, b) BM@PLC3, c) PLC3, d) EGF, e) BM, and f) control; scale bar: 100 μm. **(B)** CCK-8 for blank hydrogels. **(C)** CCK-8 for hydrogels loaded with drugs. **(D)** Hemolysis test on a) PLC3, b) PLC3-1, c) PLC3-2, d) PLC3-3, e) BM@PLC3, f) EGF@PLC3, g) Ultra-pure water, and h) saline. **(E)** The quantitative result of hemolysis test. **(F)** Cell scratch test on a) EGF@PLC3, b) EGF, c) PLC3, and d) control groups. **(G)** Measuring analysis on cell scratch test. With the data shown as means ± SD (*n* = 3).

Live/Dead staining showed that live cells (green) in each group were in the majority, and red cells were rare, even none (Figure 5A), indicating the benign biocompatibility of PLC. Besides, the result of CCK-8 for PLC suggested that the relative cell viabilities cultured with blank hydrogels were all higher than 88% (Figure 5B), revealing that PLC hardly had cytotoxicity, and were quite safe for humans. In addition, the relative cell viability of BM@PLC3 and EGF@PLC3 (Figure 5C) was higher than 88 and 100%, respectively, suggesting that (BM/EGF)@PLC not only had satisfied biocompatibility but also had a cell proliferation–promoting ability because EGF had the ability to promote L929 transport and proliferation.

The result of hemocompatibility clearly exhibited that the supernatant color of hydrogel groups had significant differences when compared with ultrapure water (positive group), while they were the same as saline groups (Figure 5D). From Figure 5E, the hemolysis ratio of each hydrogel group was lower than 5%, and the hemoglobin absorbance value was similar to the saline group or even a little lower than that, and it was demonstrated that PLC possessed excellent hemocompatibility, which could be applied in the biomedicine field. The reason that the hemoglobin absorbance value of each hydrogel group was a little lower than the saline group may be due to the addition of ε-PL. As we know, ε-PL was rich in cations and had good penetration into biofilms as a drug carrier, which could avoid something to damage cells. Besides, it was found that the cell membrane adsorption of ε-PL will reduce the critical voltage of breakage. Therefore, we could infer that with the addition of ε-PL, the relative hemolysis ratios of hydrogel groups were similar to those of the saline group or even a little lower than that, exhibiting the excellent biocompatibility of PLC.

Cell scratch experiment was carried out to evaluate the cell proliferation–promoting ability of PLC. The result (Figures 5F,G) showed that the area of cell scratch was decreased gradually on each group. At 48 h, the migration rates of EGF@PLC3, EGF, PLC3, and EGF@PLC3 groups were 70.2, 54.2, 62.7, and 48%, respectively, indicating that EGF@PLC3 had more excellent promotion of cell proliferation ability among these groups.

### Wound Healing Enhancement by (BM/EGF)@PLC *In Vivo*

**FIGURE 6:**
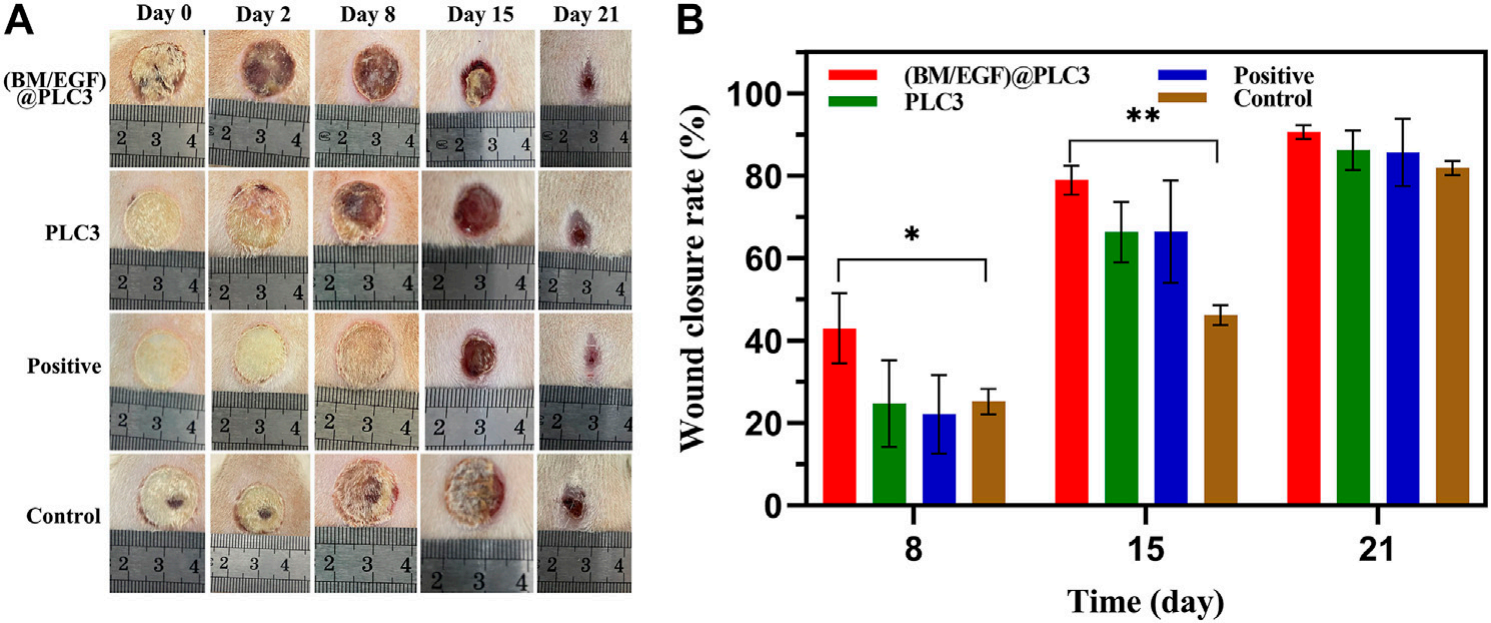
*In vivo* treatment of the burning rat model. **(A)** Assessment of the effect of treatment on days 0, 2, 8, 15, and 21. **(B)** Wound closure rates on days 8, 15, and 21 before treatment. With the data shown as means ± SD (*n* = 3, *p < 0.05, **p < 0.01).

During the treatment for 21 days, the deep-partial thickness skin burn wounds were healing gradually. The wound status of all groups declared that with the treatment of (BM/EGF)@PLC3, the necrotic tissue was eliminated and the remnant dermis was disclosed on day 2, while the necrotic tissues treated with PLC3 and sulfadiazine silver cream were shed gradually on day 8 and day 15, respectively. As for the control group, the necrotic tissue was cleaned up inapparently after 15 days (Figure 6A). Besides, wound closure rates of the (BM/EGF)@PLC3 group were achieved as 90.60% (Figure 6B), exhibiting the marvelous effect of healing on burn wound. Hence, the result demonstrated comparing the “sequential therapy,” the (BM/EGF)@PLC3 hydrogel was proven to fulfill the demands of deep burn injuries in wound cleaning and healing.

### Histopathological Analysis

**FIGURE 7:**
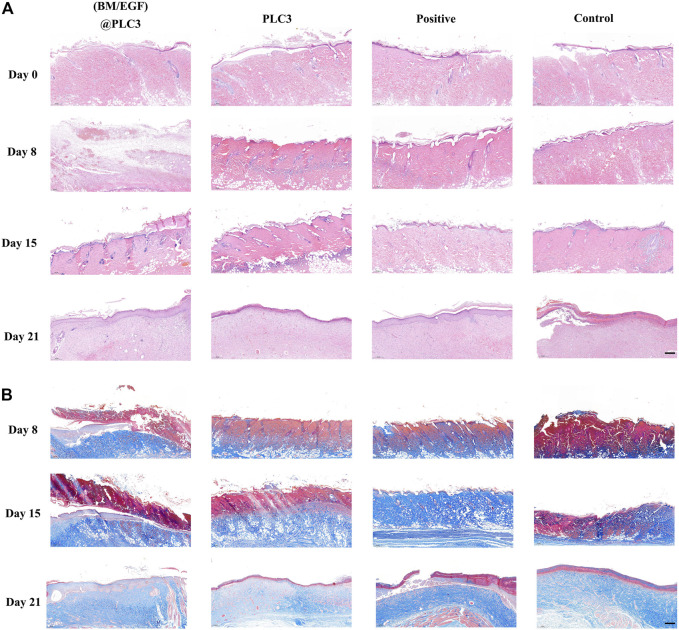
The result of histopathological examination. **(A)** H&E staining of sampled tissue on days 0, 8, 15, and 21. **(B)** Masson’s trichrome staining of samples on days 8, 15, and 21; scale bar: 200 µm.

The result of the H&E staining showed that the epidermis of wound tissue in four groups were thinner or even disappeared with skin appendages damaged partially after being scalded on day 0, indicating the successful construction of deep partial-thickness skin burn models. On day 21, the thickness of the epidermis in the (BM/EGF)@PLC3 group and positive group was thicker than that of the PLC3 group and control group (Figure 7A). The result of Masson’s trichrome staining showed that the array of collagen fiber in the (BM/EGF)@PLC3 group was orderly, and it was hard to find any cellulous on the epidermal, while there were cellulous stained red in other groups on day 21 (Figure 7B).

**FIGURE 8:**
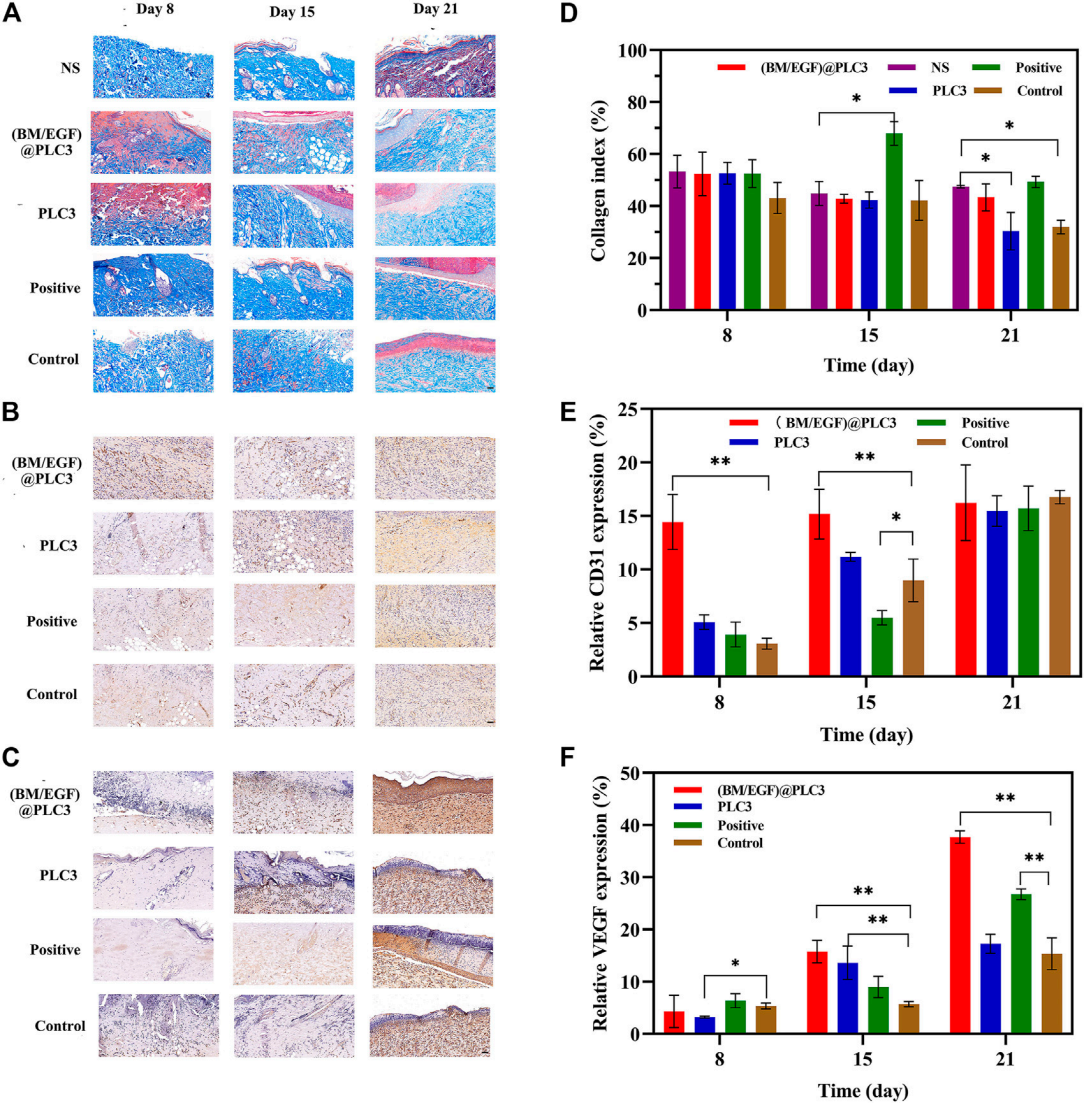
The result of quantitative detection. **(A)** Masson’s trichrome staining, **(B)** CD31 Staining, and **(C)** VEGF staining of sampled tissue on days 8, 15, and 21 before treatment. Quantitative detection for **(D)** collagen, **(E)** CD31, and **(F)** VEGF of sampled tissue. With the data shown as means ± SD (*n* = 3, *p < 0.05, **p < 0.01), scale bar: 50 µm.

Quantitative detection of collagen, CD31, and VEGF were also adopted to confirm the effect of (BM/EGF)@PLC3. The collagen index of (BM/EGF)@PLC3 group had a similar trend as the normal skin tissue (NS), while PLC3 and control groups had significant differences with NS (*p* < 0.05) on day 21 (Figures 8A,D). What is more, the proportion of CD31 in the (BM/EGF)@PLC3 group (14.44%) was nearly 5 times higher than that of the control group (3.06%) on day 8 and about double on day 15 (15.17 vs. 8.97%) (Figures 8B,E). As for the proportion of VEGF (Figures 8C,F), significant differences between the (BM/EGF)@PLC3 group and the control group were shown clearly (*p* < 0.01). The results further confirmed that (BM/EGF)@PLC3 could promote burn wound healing effectively.

### The Effect of Debridement With Bromelain

**FIGURE 9:**
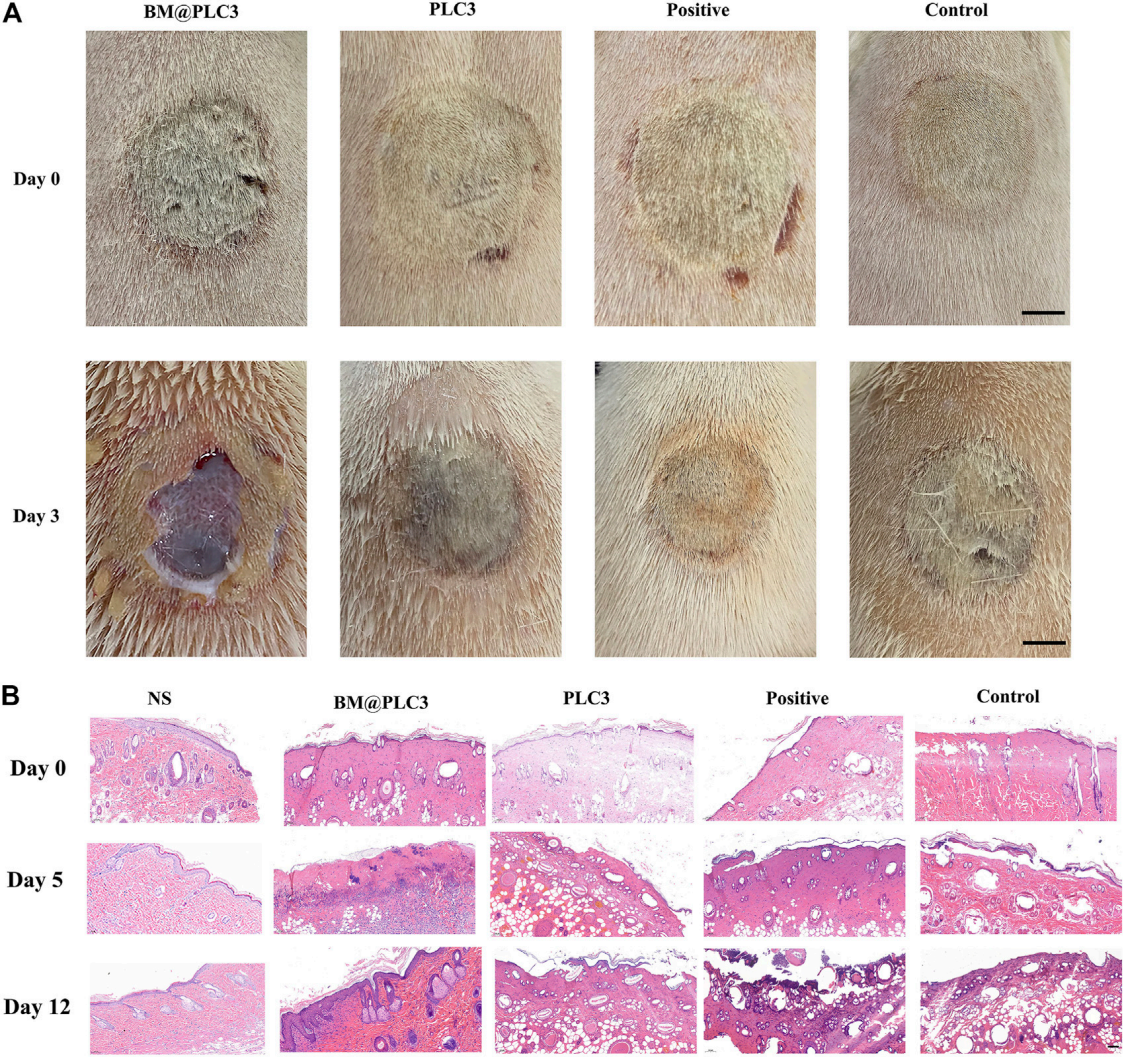
The result of burn magnification test. **(A)** The image of burn wound before treatment on days 0 and 3, scale bar: 1 cm. **(B)** H&E staining on the normal skin group (NS), BM@PLC3 group, PLC3 group, positive group, and control group on days 0, 5, and 12; scale bar: 100 μm.

After treatment for 2 days, the eschar of full-thickness skin burn wounds could be easily cleaned by tweezers only in the BM@PLC3 group (Figure 9A), revealing that bromelain had the marvelous effect to ease the eschar removing and it did not affect normal skin tissues at the same time. The H&E staining was adopted to observe the pathological changes of wounds on days 0, 5, and 12. From the result (Figure 9B), the epidermis of sample tissues became thinner and even disappeared compared with normal skin tissue (NS), and the skin appendages such as hair follicles were damaged seriously on day 0, indicating that the full-thickness skin burn wound model was successfully manufactured. On day 5, partial necrotic tissue in the BM@PLC3 group started to fall off naturally, and there were almost no eschar remaining on day 12. Moreover, the newborn epidermis was in good condition, and the skin appendages returned to normal. In contrast, the other three groups still showed inapparent wound cleaning on day 12, blocking the growth of new epidermis.

## Conclusion

In this study, a novel PLC hydrogel has been developed as a multifunctional dressing for deep burn injuries. This triple-network structure endows PLC benign mechanical property, self-healing ability, tissue-adhesiveness, antibacterial activity, biosafety, and marvelous wound cleaning and healing efficiency. In short, the (BM/EGF)@PLC hydrogel we designed has a great potential in treating deep burn injuries, and it is especially geared for irregular shaped deep burn wounds in poor medical conditions. Thus, it makes great sense in developing new-generation dressing and treatment for burn injuries in clinics.

## Data Availability

The original contributions presented in the study are included in the article/Supplementary Material; further inquiries can be directed to the corresponding authors.
