# Supplementary material for: A Sequential Therapeutic Hydrogel With Injectability and Antibacterial Activity for Deep Burn Wounds’ Cleaning and Healing
Source: Front Bioeng Biotechnol. 2021 Dec 2;9:794769. doi: 10.3389/fbioe.2021.794769 (PMC8675388; doi:10.3389/fbioe.2021.794769)
Supplement: Supplementary file 1 [file DataSheet1.docx]

Supplementary Material

# Synthetic Methods

Preparation of PF127-CHO

PF127 modified with sulfite group was synthesized according to a reference (Ziyi, et al., 2018) and the synthetic route was shown in **Supplementary Figure 1A**. Briefly, 80 mL dichloromethane and 2 mL triethylamine were added into dried PF127 (20.0 g) under ice bath. Then the mixture of 0.75 mL ethyl sulfonyl chloride and 5 mL dichloromethane were added into slowly under the protection of N_2_, and the solution was kept stirring for 24 hours at room temperature. The product was collected with dichloromethane and concentration before precipitated with diethyl ether. PF127-SO_3_ was obtained after vacuum drying.

PF127-SO_3_ obtained was dissolved by N, N-Dime ethyl formamide (DMF, 100 mL), then added into potassium carbonate (1.326 g) and p-hydroxy benzaldehyde (0.836 g). The mixture was stirred for 48 hours at 80 ℃. The product was collected with dichloromethane and concentration. Finally, the condensed mixture was precipitated with diethyl ether to obtain PF127-CHO after vacuum drying.

**Preparation of PVA-SH**

PVA was modified by esterification reaction with GSH and L-Cys as previously described (Schanuel, et al.,2015) and the synthetic route was shown in **Supplementary Figure 1B**. To be brief, PVA aqueous solution (10% w/v), GSH aqueous solution (8.4% w/v) and L-Cys aqueous solution (2.75% w/v) were mixed and stirred for 3 hours at 100 ℃ with the protection of N_2_ under reflux, and PVA-SH was obtained as a transparent liquid.

**Preparation of PLC**

PLC was prepared as the route shown on **Supplementary Figure 1C**. Firstly, ε-PL was added into PVA-SH solution, and the system was stirred well to obtain the PVA-SH/ ε-PL mixed solution. Then, PF127-CHO solution (33% w/v) was added into PVA-SH/ε-PL, with the molar ratio of amino groups in PVA-SH and aldehyde groups in PF127-CHO as 2:1, 3:1, 4:1 or 5:1, respectively, in order to investigate the influence of their different molar ratios on obtained hydrogels. The mixture was stirred incessantly to make it gelatinize, forming a series of PLCs named PLC2, PLC3, PLC4, PLC5, respectively **(****Supplementary Figure 2A)**. Therein the ratio of 3:1 (PLC3) was proved to be the suitable functionality ratio between amino and aldehyde groups in following experiments to research the effect of different ε-PL content on hydrogels. Similar synthetic pathways were conducted, except for the molar ratio of hydroxy groups in PVA and carboxyl groups in ε-PL was changed from 75:1 (PLC3) to 50:1, 100:1 and 200:1, respectively. Then a series of hydrogels with different ε-PL content were formed and named PLC3-1(50:1), PLC3-2 (100:1) and PLC3-3 (200:1), respectively **(Supplementary Figure 2B)**.

**Preparation of (BM/EGF)@PLC3 Hydrogel**

Among these PLCs (PLC3, PLC3-1, PLC3-2 and PLC3-3), we chose PLC3 to load drugs (bromelain and EGF) for the following researches. Bromelain (20μg/mL) and EGF (10μg/mL) were added into hydrogel respectively. The obtained system was named (BM/EGF)@PLC3 briefly.

**2 The Results of Characterization**

**The Results of ^1^H NMR and FT-IR Characterization**

The ^1^H NMR spectra of PF127-CHO was shown in **Supplementary Figure 3A**. The ^1^H NMR spectra comparison of PF127, PF127-SO_3_ and PF127-CHO was provided in **Supplementary Figure 3B**. As the results showed, the appearance of chemical shift (δ=9.87, 9.77) belonged to aldehyde groups, indicating the successful synthesis of PF127-CHO. PVA-SH was verified by ^1^H NMR **(Supplementary Figure 3C)**, and the comparison of ^1^H NMR between PVA and PVA-SH was provided in **Supplementary Figure 3D**. Also, the appearance of chemical shift (δ=8.67) belonged to amino groups, indicating that the successful synthesis of PVA-SH.

FT-IR was performed to further confirm the formation of PLC through characterization of special peaks **(Supplementary Figure 4A)**. For the polymer PF127-CHO, the wavenumber of 1692.29 cm^-1^ and 843.56 cm^-1^ belonged to the vibration of aldehyde groups. For PVA-SH/ɛ-PL, the wavenumbers of 3069.14 cm^-1^, 2930.60 cm^-1^, 1671.43 cm^-1^ and 1319.79 cm^-1^ were affiliated to the vibration of carboxyl groups. For PLC3 hydrogel, the wavenumbers of 1679.60 cm^-1^ and 1602.57 cm^-1^ belonged to amide groups, demonstrating the successful synthesis of PLC3.

**The Results of Rheological Tests of PLC2, PLC3 and PLC4**

The shear stress of PLC2, PLC3 and PLC4 were tested by rheological tests **(Supplementary Figure 4B)**. According to the results, PLC2 possessed the highest shear stress among these three samples, corresponding to the weakest shear thinning property. Therefore, compared to PLC2, PLC3 and PLC4 had the better performance in shear stress. However, PLC4 didn’t crosslink to form hydrogel in 1 minute while PLC2 and PLC3 could gel under stirring quickly, suggesting the gelling time of PLC4 was longer than PLC2 and PLC3. Thus, PLC3 was the most suitable system for subsequent animal experiments. The molar ratio of amino groups and aldehyde groups (3:1) during its preparation was determined as the optimal selection, and was adopted in the synthesis of PLC3-1, PLC3-2 and PLC3-3.

**3 Supplementary Figures**


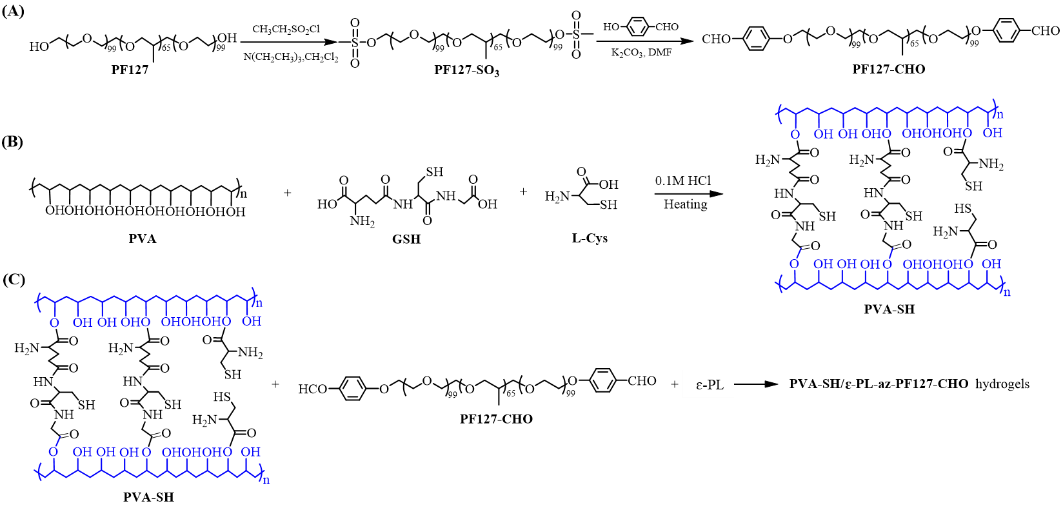


**Supplementary Figure 1.** Synthetic route of (A) benzaldehyde-terminated PF127 (PF127-CHO), (B) PVA modified with sulfhydryl and amino groups (PVA-SH) and (C) PVA-SH/ɛ-PL-az-PF127-CHO hydrogels (PLC).

**
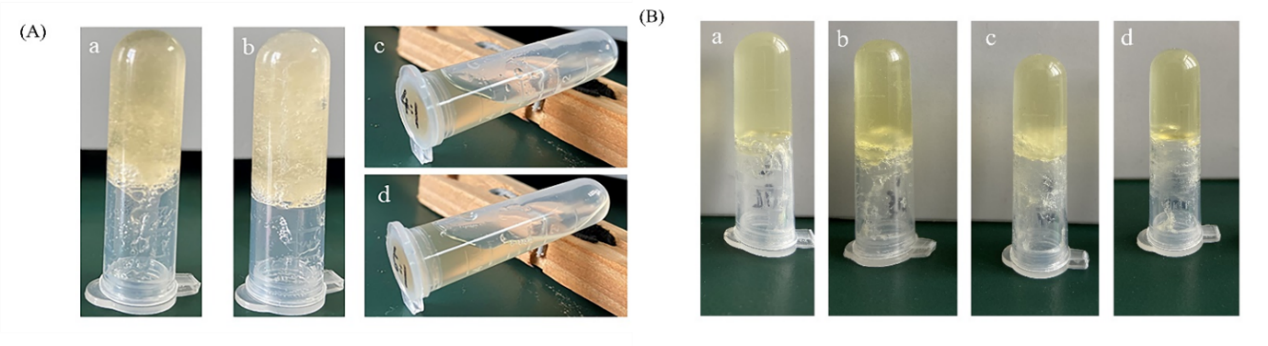
**

**Supplementary Figure 2.** Preparation of PVA-SH/ɛ-PL-az-PF127-CHO hydrogels (PLC) for (A) a) PLC2, b) PLC3, c) PLC4, d) PLC5 and (B) a) PLC3, b) PLC3-1, c) PLC3-2 and d) PLC3-3.


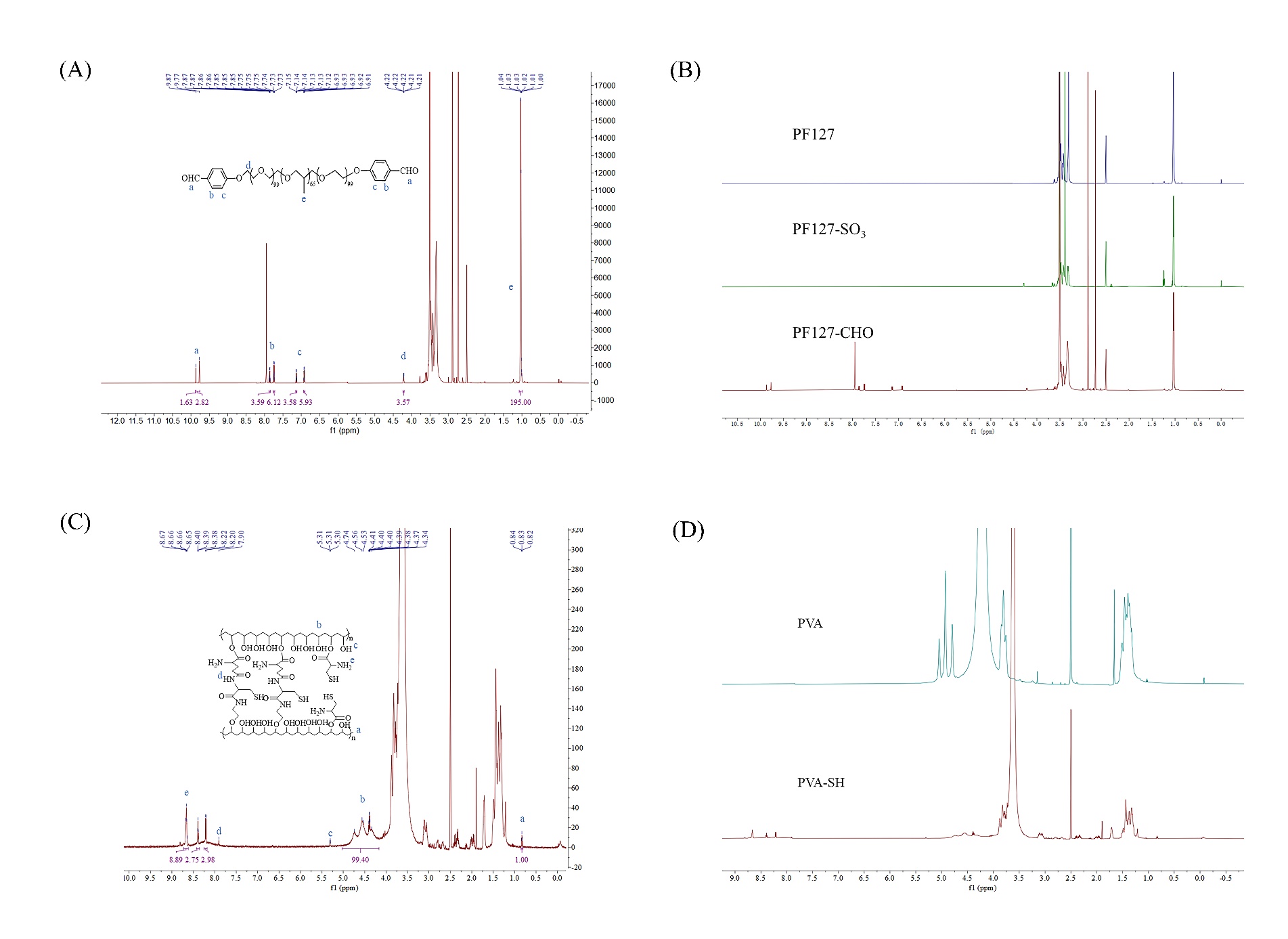


**Supplementary Figure 3.** (A) ^1^H NMR spectrum of PF127-CHO (DMSO, 600 MHz). (B) The comparison of ^1^H NMR spectrum for PF127, PF127-SO3 and PF127-CHO (DMSO, 600 MHz). (C) ^1^H NMR spectrum of PVA-SH (DMSO, 600 MHz).


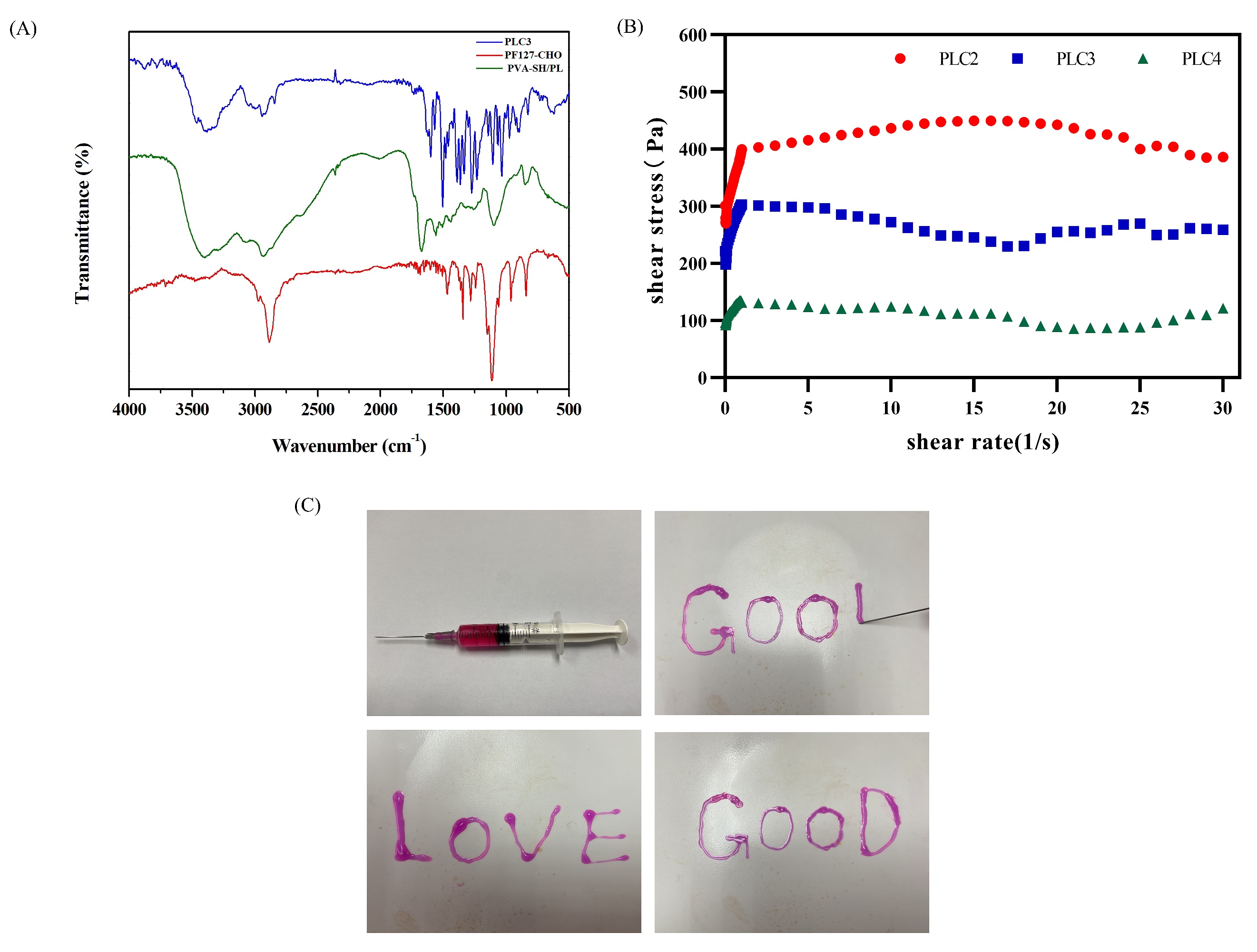


**Supplementary Figure 4.** (A) FT-IR test of PLC3, PF127-CHO and PVA-SH/ɛ-PL. (B) Rheological tests of PLC2, PLC3 and PLC4. (C) Microscopic phenomenon of injectable and thermal-responsive hydrogel.
